# Minimally invasive 1 mm skin biopsies capture site-specific transcriptomic heterogeneity in vitiligo

**DOI:** 10.3389/fimmu.2026.1829996

**Published:** 2026-05-14

**Authors:** Danique Berrevoet, Arno Belpaire, Elise Van Caelenberg, Barbara Boone, Guillaume Cattebeke, Yannick Gansemans, Elise Callens, Koen Deserranno, Dieter Deforce, Reinhart Speeckaert, Filip Van Nieuwerburgh

**Affiliations:** 1 Laboratory of Pharmaceutical Biotechnology, Faculty of Pharmaceutical Sciences, Ghent University, Ghent, Belgium; 2 Department of Dermatology, Ghent University Hospital, Ghent, Belgium

**Keywords:** bulk RNA sequencing, minimally invasive, skin biopsies, therapeutic repurposing, transcriptomics, vitiligo

## Abstract

**Background:**

Vitiligo is a chronic inflammatory skin disease characterized by clinical and molecular heterogeneity across lesional, perilesional and non-lesional skin within the same individual. Understanding these region-specific differences is essential for identifying early disease processes and developing targeted therapeutic strategies. Skin biopsies represent a key approach to explore these differences, yet conventional 3–5 mm biopsies are invasive, requiring sutures, extended healing time and leaving visible scarring.

**Objectives:**

The objective of this study was to characterize region-specific transcriptomic alterations across lesional, perilesional, and non-lesional skin in vitiligo, using minimally invasive 1 mm skin punch biopsies.

**Methods:**

In this study, bulk RNA sequencing was performed on 105 skin biopsies obtained from perilesional and (non-)lesional skin of non-segmental vitiligo patients, as well as healthy control skin. Differential gene expression was followed by pathway-level analyses, and Connectivity Map–based perturbational profiling was performed to predict candidate therapeutic compounds capable of reversing the lesional transcriptional signature.

**Results:**

Transcriptomic profiling of 1 mm biopsies revealed disease-associated changes across lesional, perilesional, and non-lesional vitiligo skin, with distinct region-specific gene signatures reflecting immune activation and metabolic reprogramming. Pathway analysis further identified dysregulation of both canonical and underexplored pathways, including NOD-like receptor signaling and neutrophil extracellular trap formation, alongside altered cellular clearance mechanisms potentially implicated in lesion persistence. Connectivity Map analysis nominated compounds predicted to reverse the lesional transcriptional signature, spanning epigenetic regulators, tyrosine kinase inhibitors, and metabolic modulators.

**Conclusion:**

This approach uncovers potentially pathogenic pathways contributing to vitiligo pathogenesis and provides a translational framework for therapeutic hypothesis generation and future clinical studies.

## 1 Introduction

Vitiligo is the most common skin depigmentation disorder worldwide and is characterized by selective loss of melanocytes, resulting in depigmented lesions (1). Substantial progress has been made in elucidating its pathogenesis, leading to its classification as an autoimmune disorder. Central to this understanding is the identification of immunological mechanisms in which melanocyte-specific cytotoxic T cells mediate targeted melanocyte destruction, a process in which the Janus Kinase/Signal Transducer and Activator of Transcription (JAK-STAT) signaling pathway plays a critical role (2). While these insights have facilitated the development of promising targeted therapies such as Janus kinase (JAK) inhibitors, currently available treatments are still limited by side effects, inconsistent efficacy, and high relapse rates upon discontinuation (2–4). This variability in treatment response may be partly attributed to the involvement of alternative or additional immune pathways beyond the well-characterized JAK-STAT signaling axis, operating within the local skin microenvironment. Defining these lesion-resident pathways can inform mechanism-based treatments, highlighting agents predicted to correct disease-associated states.

Transcriptomic profiling of skin offers a high-resolution approach to uncover local immune and metabolic changes, yet its routine application in dermatology is constrained by the invasiveness of standard 3–5 mm punch biopsies, which induce scars, require suturing, and are impractical for repeated sampling or routine clinical use (5, 6). While other non-invasive approaches such as tape-stripping have been explored, they are limited to the superficial layers of the epidermis and fail to capture the full cellular complexity of the skin microenvironment (7). Moreover, limited studies have systematically compared lesional, perilesional, and non-lesional skin from the same vitiligo patient using transcriptomic approaches, limiting our understanding of disease heterogeneity within individuals. Emerging evidence suggests that vitiligo extends beyond visibly depigmented lesions. Perilesional skin, representing the transition between lesional and visually unaffected areas, exhibits active immune infiltration, while non-lesional skin may harbor subclinical inflammatory or metabolic alterations indicative of a prelesional state (8–10). This spatial heterogeneity highlights the importance of region-specific molecular profiling to delineate the continuum of disease activity within individual patients.

In this study, we apply bulk RNA sequencing to 1 mm punch biopsies from lesional, perilesional, non-lesional, and control skin to define region-specific transcriptional signatures that capture both established and previously underrecognized disease pathways and to evaluate the feasibility and biological fidelity of minimally invasive transcriptomics. Moreover, our approach aims to explore candidate pharmacologic modulators capable of reversing the vitiligo-associated expression profile. Through this framework, we seek to expand current understanding of vitiligo pathogenesis while highlighting the translational potential of small-biopsy transcriptomics as a practical and scalable tool for molecular discovery and translational research in vitiligo and other skin diseases.

## 2 Materials and methods

### 2.1 Patient inclusion

A total of 28 patients with non-segmental vitiligo and 27 healthy controls were recruited between July 2024 and May 2025 at the Department of Dermatology, Ghent University Hospital. The study was approved by the Ethics Committee of Ghent University Hospital, and all participants provided written informed consent. Inclusion criteria comprised a confirmed diagnosis of non-segmental vitiligo, age ≥18 years, and the ability to consent. Individuals with segmental vitiligo or concurrent immune-mediated inflammatory disorders were excluded because of their potential confounding influence on the analyses. Study size was determined by participant availability during the recruitment period.

### 2.2 Study design and clinical assessment

Transcriptomic profiles were generated from all collected skin biopsies. Skin region (lesional, perilesional, non-lesional, and control skin) was used for comparative analyses. Disease activity was assessed using standardized follow-up photographs and quantified with the Vitiligo Disease Activity Score (VDAS) and Vitiligo Disease Improvement Score (VDIS) (11). Patients were classified as having active disease if objectively evaluated before-and-after photographs demonstrated an increase in VDAS score, typically assessed over a 6-month interval (maximum of 12 months). In the absence of photographic documentation (e.g. at the initial consultation), active disease was determined based on a self-reported mVIDA score indicating disease activity within the preceding 3 months.

### 2.3 Sample collection

**Figure 1:**
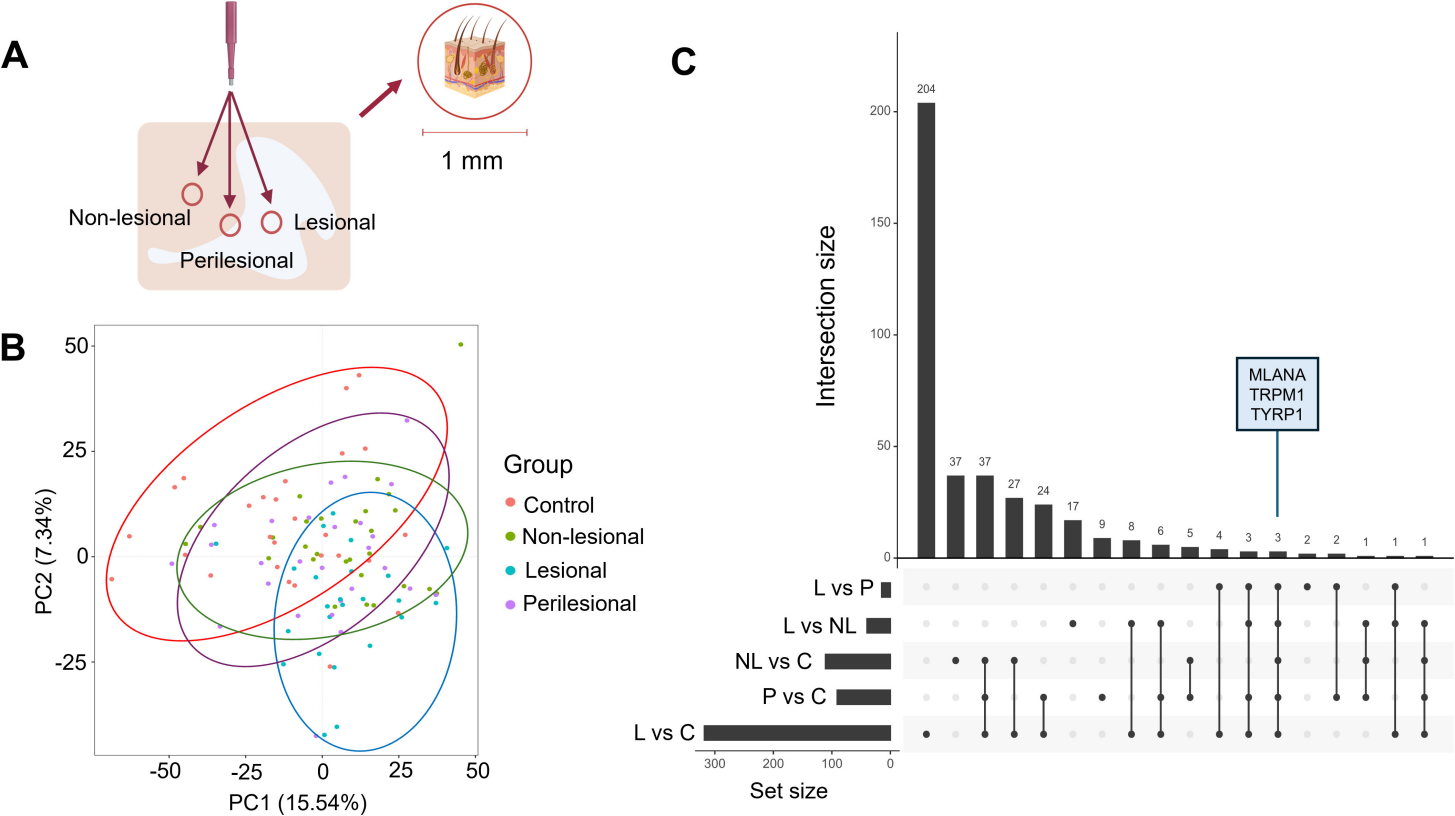
Shared and distinct transcriptional alterations across vitiligo skin regions. **(A)** Schematic representation of the biopsy sampling strategy across lesional, perilesional, and non-lesional vitiligo skin. Created in BioRender. Deforce, D. (2026) https://BioRender.com/rc3ftoz. **(B)** PCA of transcriptomic profiles from lesional, perilesional and non-lesional skin from vitiligo patients, and skin samples from healthy controls. **(C)** UpSet plot showing overlapping differentially expressed genes (DEGs) across comparisons. The set size represents the total number of DEGs identified in each individual comparison, while the intersection size indicates the number of DEGs shared between different comparisons, as indicated by the connected dots below the bars. This visualization highlights both unique and shared transcriptional changes across skin regions in vitiligo. The perilesional versus non-lesional comparison is excluded, as no significant DEGs were detected in this comparison (|log_2_FC|≥1, P_adj_ < 0.05). L, lesional skin; P, perilesional skin; NL, non-lesional skin; C, control skin.

Skin punch biopsies (diameter: 1 mm) were obtained under local anaesthesia (xylocaine 2% + adrenaline) using sterile punch tools (Kai Europe GmbH, Germany). Where feasible, each patient contributed lesional, perilesional, and non-lesional skin biopsies to minimize inter-individual variability. Lesional skin referred to depigmented skin sampled centrally within the vitiligo lesion. Perilesional skin was defined as a biopsy centered on the clinically visible lesion border, such that the sampled area corresponded to the transition zone between depigmented and unaffected skin. Non-lesional skin was defined as clinically unaffected skin sampled 1 cm from the visible lesion border, showing no apparent depigmentation (**Figure 1A**). In total, 24 lesional, 27 perilesional and 27 non-lesional skin biopsies were collected from 28 patients. The punch biopsies were preferentially taken from the arm or leg to ensure consistency in anatomical location. Healthy controls (n = 27) each contributed one biopsy, collected from the upper arm. All biopsies were immediately snap frozen in liquid nitrogen and stored at −80 °C until further processing.

### 2.4 Sample processing

Total RNA was extracted from 1 mm skin punch biopsies using the TRIzol/chloroform method. The tissue was lysed mechanically using a 1000 µL pipette tip, and sheared through a syringe (Terumo Corporation) fitted with a 20Gx1½" needle (Terumo Corporation). To minimise RNA degradation, SuperaseIn RNase Inhibitor (Thermo Fisher Scientific) was added. This was followed by column-based RNA purification with on-column DNase treatment (Direct-zol Microprep, Zymo Research). All samples underwent an additional DNase treatment (TURBO DNA-*free*™ Kit, Invitrogen). RNA concentration was measured using the Qubit^®^ 2.0 Fluorometer with Qubit RNA HS Assay Kit (Thermo Fisher Scientific), and RNA integrity was assessed using the 5200 Fragment Analyzer (Agilent Technologies). Sequencing libraries were generated using the QuantSeq 3′ mRNA-Seq V2 Library Prep Kit (Lexogen). Healthy control samples were processed in parallel using identical procedures. RNA quantity and integrity from all 1 mm samples consistently met library preparation requirements.

### 2.5 RNA sequencing and pre-processing

Bulk RNA sequencing of all 105 samples was performed on the AVITI™ Cloudbreak Freestyle platform (high-output kit), generating 75 bp single-end reads. The biopsies were processed in two library-preparation batches and sequenced across two runs. Raw reads were assessed for quality and length using FastQC (v0.12.1). Putative contaminations were evaluated with FastQ Screen (v0.15.3). Adapter trimming was performed using cutadapt (v5.0), with filtering to remove reads containing ambiguities or reads below phred 20. Trimmed read pairs were re-evaluated using FastQC. Alignment against the human reference genome (GRCh38, ENSEMBL release 112) was performed using STAR (v2.7.11b). UMI-based deduplication of mapped reads was performed with UMI-tools (v1.1.6), and feature counting at the gene and transcript isoform level was done using rsem-calculate-expression (RSEM; v1.3.1). The gene-level counts were used for all downstream analyses in R (v4.5.0).

### 2.6 Protein-coding gene detection and clustering analysis

Gene annotations were imported from the reference GTF via rtracklayer (v1.68.0) and integrated with the obtained counts using dplyr (v1.1.4) and tidyr (v1.3.1). To quantify detected protein-coding genes, only entries annotated as protein_coding in the GTF were retained. For each sample, detected genes (counts > 0) were tallied per biotype, and biotype composition was expressed as the percentage of detected genes belonging to each biotype. Principal component analysis (PCA) was performed on the rlog-transformed (DESeq2; v1.48.1) counts using prcomp to assess sample clustering and batch effects across all samples.

### 2.7 Differential expression analysis

Differential gene expression analysis was conducted using EdgeR (v4.2.2) (12). Feature counts were TMM-normalized and correction of the P values for repeated testing (adjusted P value (P_adj_)) was performed using the Benjamini-Hochberg method. Genes with P_adj_ < 0.05 and |log_2_FC|≥1 (≥2-fold) were considered significantly differentially expressed. Samples were classified as lesional, perilesional, non-lesional, or control. Tested contrasts: lesional vs control, perilesional vs control, non-lesional vs control, lesional vs perilesional, lesional vs non-lesional, and perilesional vs non-lesional. For within-patient comparisons between skin regions (lesional, perilesional, and non-lesional), repeated measures were handled in edgeR using a paired design with patient_id included as a blocking factor in the design matrix: design <- model.matrix(~patient_id + group). Results are reported as Group 1 vs Group 2, with positive log_2_FC indicating higher expression in Group 1. Gene symbols follow HGNC nomenclature.

### 2.8 Gene set enrichment analysis

Gene Set Enrichment Analysis (GSEA) was performed using clusterprofiler on the full ranked (logFC) gene lists for each contrast (13). Analyses were conducted using annotated gene sets from the Kyoto Encyclopedia of Genes and Genomes (KEGG; release 116.0*)* and Gene Ontology (GO; v3.21.0) databases. Enriched pathways were considered significant if P_adj_ < 0.05.

### 2.9 Connectivity map analysis

Connectivity Map (CMap) queries were run on the CLUE platform (with BING extension) using the lesional vs control differentially expressed gene (DEG) set. Genes were ranked by a composite of log_2_FC and P_adj_ and split into up- and down-regulated lists. Only DEGs with |log_2_FC| ≥ 0.585 (≥1.5-fold) and P_adj_ < 0.05 were eligible. As CMap queries accept ≤150 genes per direction, genes outside the L1000+BING universe were replaced by the next highest-ranked DEGs (ranked by |log_2_FC| × −log_10_ (P_adj_)), and if >150 valid genes were available, the top 150 were used. Analyses were restricted to small-molecule perturbations (trt_cp) in the A375 (melanocytic) cell line. Similarity was scored by the raw connectivity score (raw_cs) (positive = similar; negative = inverse). Significance used fdr_q_nlog10 and high-confidence hits were defined as fdr_q_nlog10 ≥ 15.35 (q < 10^-15·35^).

## 3 Results

### 3.1 Participant characteristics

This cross-sectional study used 105–1 mm skin punch biopsies from 28 patients with non-segmental vitiligo and 27 healthy controls. Participant characteristics are summarized in **Table 1**. All biopsies met the minimum RNA input requirement (10 ng total RNA) suitable for library preparation. The cohort represented a balanced sex distribution (50% female) with a median age of 51 years (IQR 36–59) and median disease onset at 35 years. Sixteen patients (57%) exhibited clinically active disease based on standardized VDAS/VDIS and mVIDA assessments.

**Table 1:** Participant characteristics.

Characteristic	Vitiligo patients (n = 28)	Healthy controls (n = 27)
Age (years), median [IQR]	51 [36–58.5]	32 [27–45]
Sex (female/male)	14/14	20/7
BMI (kg/m^2^), mean ± SD	24.7 ± 3.5^a^	22.6 ± 4.1
Age of onset vitiligo (years), median [IQR]	35 [21–50]	NA
Family history (1st-degree relative), n/N (%)	3/26 (11.5%)^b^	NA
Current treatment, n (%)	Topical: 17 (60.7%), Methotrexate (MTX): 7 (25.0%), None: 4 (14.3%)	NA
Current smokers, n/N (%)	2/28 (7.1%)	3/27 (11%)
Disease status: active, n/N (%) Disease status: stable, n/N (%)	16/28 (57.1%)^c^ 12/28 (42.9%)^d^	NA
VES score (%), median [IQR]	4.26 [2.35–7.74]	NA
VDAS_15_ ≥ 1, n/N (%) VDAS_15_ = 0, n/N (%) VDIS_15_ ≥ 1, n/N (%) VDIS_15_ = 0, n/N (%)	12/18 (66.7%)^e^, 6/18 (33.3%) ^e^, 1/18 (5.6%) ^e^, 17/18 (94.4%)^e^	NA
mVIDA score <3 months, n/N (%) mVIDA score 3–6 months, n/N (%) mVIDA score 6–12 months, n/N (%) mVIDA score > 1 year, n/N (%)	14/28 (50.0%) 1/28 (3.6%) 7/28 (25.0%) 6/28 (21.4%)	NA

^a^ BMI was unavailable for one patient.

^b^ Family history unknown for two participants; percentages are based on available data (n = 26).

^c^ Active disease defined as lesion progression or new lesions within the past 6 months.

^d^ Stable disease defined as no lesion progression or new lesions within the past 6 months.

^e^ VDAS/VDIS scores unavailable for 10 first-time consultations; percentages based on available data (n = 18).

### 3.2 Global transcriptional characterization of vitiligo skin

Bulk RNA sequencing achieved a mean of 38.7 million reads per sample, detecting approximately 15,000 protein-coding genes per biopsy (**Supplementary Figure 1**). PCA revealed distinct separation between lesional and control skin, reflecting extensive transcriptional reprogramming in established vitiligo lesions (**Figure 1B**). Perilesional and non-lesional samples showed substantial overlap and partial proximity to lesional skin, suggestive of early or subclinical disease-related activity beyond visibly affected areas.

Differential expression analysis (**Supplementary Figure 2**, panels A-F) identified 320 DEGs in lesional versus control skin (45 upregulated, 275 downregulated), 92 DEGs in perilesional versus control skin (30 upregulated, 62 downregulated), and 124 DEGs in non-lesional versus control skin (63 upregulated, 61 downregulated). Within-patient comparisons revealed 40 DEGs between lesional and non-lesional skin (4 upregulated, 36 downregulated), 15 DEGs (all upregulated) between perilesional and lesional skin, and no significant changes between perilesional and non-lesional skin (**Supplementary Table 1**). The majority of DEGs were unique to the lesional versus control comparison, reflecting the distinct transcriptional profile of lesional vitiligo skin (**Figure 1C**). Only 3 genes (*MLANA, TRPM1, TYRP1*) were common to all comparisons.

### 3.3 Minimally invasive transcriptomics captures canonical, novel and low-expression vitiligo signatures

**Figure 2:**
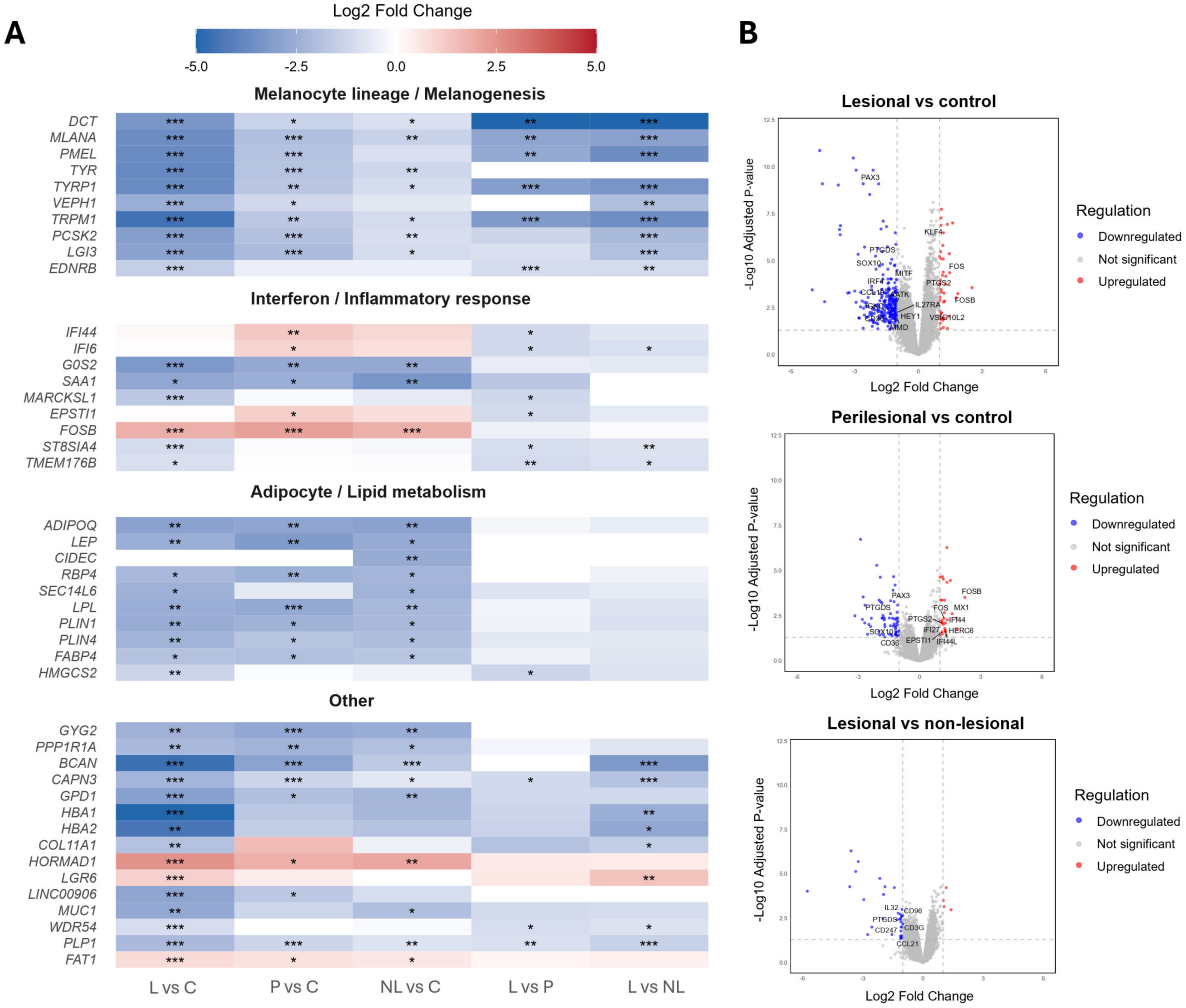
Top differentially expressed genes and immune signatures in vitiligo skin. **(A)** Combined set of top 15 DEGs across pairwise comparisons. Genes are displayed on the y-axis and grouped by functional category, based on their primary biological roles as defined in the literature. Cell color indicates the log2 fold change (blue: downregulation; red: upregulation), with statistical significance indicated by asterisks (P*_adj_* < 0.05, *; < 0.01, **; < 0.001, ***). L, lesional; P, perilesional; NL, non-lesional; C, control. **(B)** Volcano plots highlight immune-related transcriptional changes in vitiligo skin. Volcano plots display DEGs in lesional versus control, perilesional versus control, and lesional versus non-lesional skin. Immune-related genes including cytokines, chemokines, interferon-stimulated genes, transcription factors, and immune modulators, are annotated and color-coded (blue: downregulation; red: upregulation, grey: not significant).

The top differentially expressed genes across all comparisons reflected established features of vitiligo pathogenesis (**Figure 2A**). Melanocyte- and pigmentation-associated transcripts (e.g. *TYR, PMEL, DCT, PCSK2*) were markedly downregulated in lesional skin, reflecting melanocyte depletion in depigmented areas. Additionally, perilesional skin exhibited upregulation of inflammation-associated transcripts including *IFI44*, *IFI6*, and *FOSB*, consistent with active interferon signaling and early immune activation at sites of ongoing pigment loss.

Beyond canonical melanocytic and immune genes, several underexplored candidates (e.g. *PPP1R1A, FAT1, HORMAD1*) emerged among the most dysregulated transcripts, suggesting additional signaling and regulatory processes not previously implicated in vitiligo. The dataset also captured low abundant but biologically meaningful immune transcripts, including cytokine and chemokine signaling molecules (*IL27RA, IL32, IL4I1, CCL19, CCL21*), immune modulators (*CD36, CD96, CD3G, CD247*), transcription factors (*KLF4, PAX3, IRF4, MITF, FOS, FOSB, HEY1, SOX10*), and a strong type I interferon response signature (*IFI44, IFI27, IFI44L, IFI6, MX1, EPSTI1, HERC6*) (**Figure 2B**). Three comparisons are shown in **Figure 2B**; the remaining data are provided in **Supplementary Figure 3**.

### 3.4 Pathway-level dysregulation across skin regions

**Figure 3:**
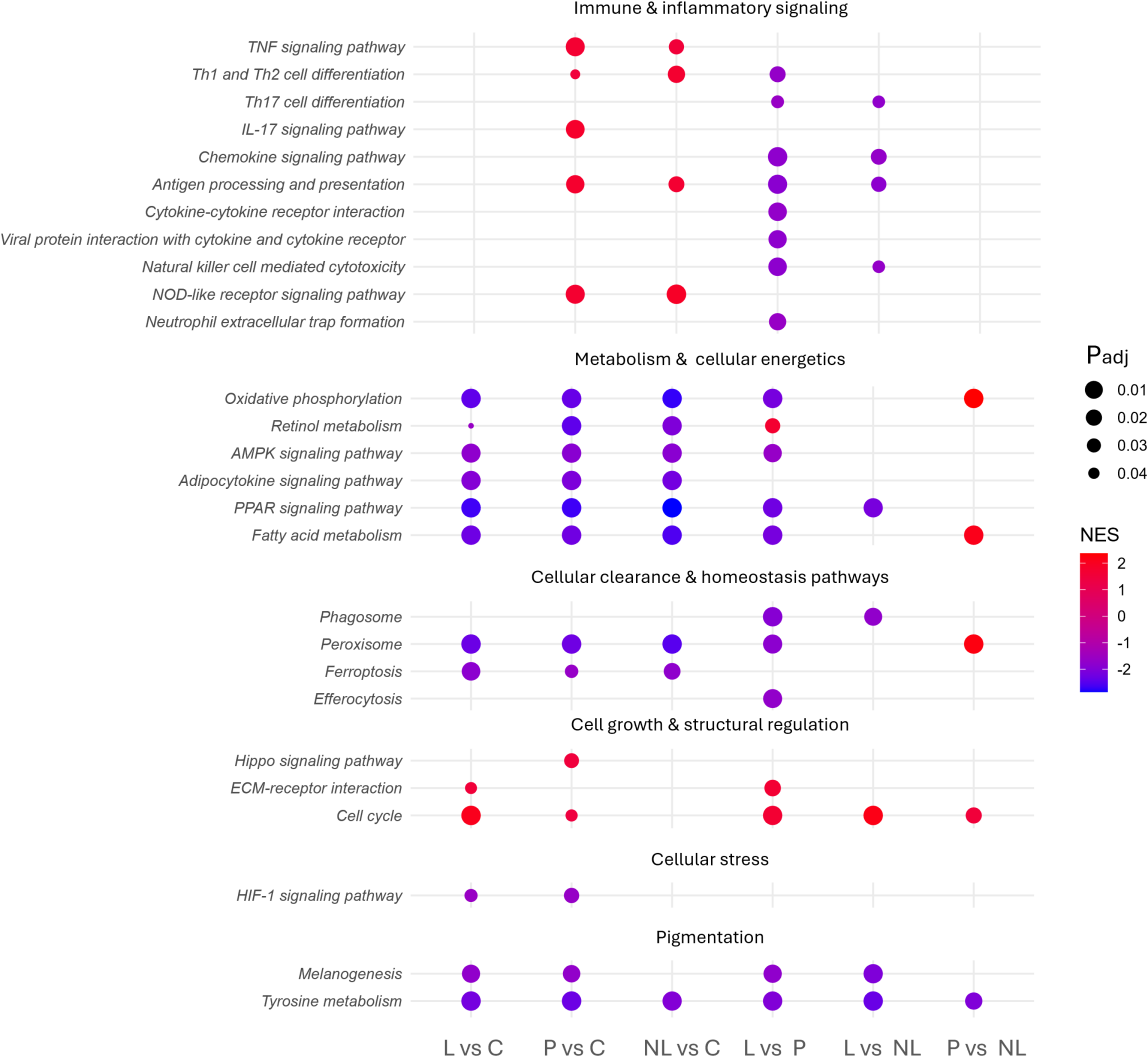
Dot plot summarizing KEGG pathways of interest significantly enriched across pairwise comparisons. Each dot denotes one KEGG pathway; the x-axis shows the indicated comparisons and the y-axis lists pathways. Dot color encodes NES (red = positive enrichment, blue = negative enrichment). Dot size reflects significance and is inversely proportional to the P_adj_ (larger dots indicate smaller P_adj_). L, lesional skin; P, perilesional skin; NL, non-lesional skin; C, control skin.

GSEA revealed consistent negative enrichment (Normalized Enrichment Score (NES) < 0; P_adj_ < 0.05) of metabolic pathways in vitiligo skin, including oxidative phosphorylation, peroxisome proliferator-activated receptor (PPAR) signaling, and fatty acid metabolism (**Figure 3**). In parallel, immune and inflammatory programs were enriched (NES > 0; P_adj_ < 0.05) in both perilesional and non-lesional skin, encompassing significant signals in tumor necrosis factor (TNF), NOD-like receptor (NLR) and T helper (Th)1/Th2 cell differentiation pathways, as well as antigen processing and presentation. Several less-studied pathways, such as phagosome, peroxisome, ferroptosis, and efferocytosis, were also significantly altered, indicating additional layers of cellular dysregulation. Additionally, “negative regulation of gene expression, epigenetic” (GO:0045814) was positively enriched in lesional versus control skin (**Supplementary Table 2**), hinting at altered chromatin-mediated transcriptional control and supporting the therapeutic relevance of epigenetic modulators identified later by CMap analysis. Full GSEA results are available in **Supplementary Table 3**.

To assess whether the 1 mm biopsy approach recapitulates established vitiligo biology in an independent dataset, a targeted external comparison was performed against a recent bulk RNA sequencing vitiligo study (**Supplementary Table 4**) (10). Specifically, both datasets showed canonical melanocyte-loss, with *TYR, DCT, PMEL, MLANA, TYRP1*, and *TRPM1* all downregulated in the lesional versus control comparison. At pathway level, both studies further converged on Th1-, Th2-, and Th17-related inflammatory programs, with this dysregulation also present in non-lesional skin in both studies.

### 3.5 Connectivity Map analysis identifies candidate signature-reversing compounds

CMap analysis of the ranked lesional versus control gene expression signature (|log_2_FC| ≥ 0.585) nominated compounds whose drug-induced expression profiles in A375 cells were inverse to the lesional vitiligo signature, thereby modeling compounds with the potential to counteract the disease-associated transcriptional state of lesional skin in vitiligo. After applying filtering criteria (trt_cp; –log_10_ FDR ≥ 15.35; A375 cell line), 170 high-confidence candidates were retained (**Supplementary Table 5**) and subsequently screened for previously reported or mechanistically plausible links to vitiligo. The major compound classes corresponded to the pathway-level abnormalities identified in our dataset, including epigenetic regulation, inflammatory and immune signaling, and metabolic dysregulation. Established vitiligo treatments such as glucocorticoids (dexamethasone (raw_cs = -0.38), hydrocortisone (raw_cs = -0.39), medrysone (raw_cs = -0.35)) and methotrexate (raw_cs = -0.35), together with the JAK2 inhibitor NVP-BSK805 (raw_cs = -0.38), were identified among the negatively correlated signatures, supporting the biological relevance of the dataset.

Beyond these reference compounds, epigenetic regulators targeting histone methylation (LLY-507, UNC-1999, BIX-01294, and GSK-343) were among the strongest inverse hits, consistent with the positive enrichment of epigenetic silencing pathways observed in lesional skin (**Supplementary Table 2**). Inhibitors of growth factor and tyrosine kinase signaling (golvatinib, cediranib, lapatinib, ARRY-334543, CGP-53353, dasatinib, SU-11274), together with the Rho-associated protein kinase (ROCK) inhibitor GSK-269962, also showed strong negative connectivity, indicating that modulation of (vascular) epidermal growth factor (VEGFR/EGFR), related non-JAK tyrosine kinase pathways, and ROCK-mediated cytoskeletal and tissue-remodeling programs may counteract disease-associated transcriptional states. In addition, immune and metabolism-linked modulators such as STA-5326, ibrutinib, PRT-062607, dipyridamole and PF-543, also demonstrated inverse transcriptional relationships, reflecting perturbation of their respective pathways; cytokine, Bruton’s tyrosine kinase (BTK), spleen tyrosine kinase (SYK), phosphodiesterase (PDE) and sphingosine kinase signaling (SphK). Collectively, these findings highlight multiple candidate therapeutic entry points converging on chromatin remodeling, immune regulation, and cellular metabolism (**Table 2**).

**Table 2:** Candidate drug repurposing compounds predicted to reverse the vitiligo transcriptional signature ranked by correlation score.

Cell line	Perturbagen name	Mode of action	Connectivity score	FDR (-log_10_)^a^
A375	LLY-507	SMYD2 inhibitor	-0.44	***
A375	Cediranib	VEGFR inhibitor	-0.44	***
A375	Golvatinib	VEGFR inhibitor	-0.43	***
A375	SU-11274	Tyrosine kinase inhibitor	-0.42	***
A375	UNC-1999	Histone lysine methyltransferase inhibitor	-0.40	***
A375	dipyridamole	Phosphodiesterase inhibitor	-0.39	***
A375	STA-5326	Interleukin inhibitor	-0.37	***
A375	ARRY-334543	EGFR inhibitor	-0.35	***
A375	CGP-53353	EGFR inhibitor	-0.39	***
A375	GSK-343	Histone lysine methyltransferase inhibitor	-0.38	***
A375	GSK-269962	ROCK inhibitor	-0.38	***
A375	BIX-01294	Histone lysine methyltransferase inhibitor	-0.36	***
A375	ibrutinib	BTK inhibitor	-0.35	***
A375	lapatinib	EGFR inhibitor	-0.35	***
A375	PRT-062607	Syk inhibitor	-0.34	***
A375	PF-543	Sphingosine kinase inhibitor	-0.34	***
A375	dasatinib	Tyrosine kinase inhibitor	-0.34	***

^a^ As per CMap conventions, FDR significance values are reported as –log_10_(q). Scores above 15.35 indicating extremely strong enrichment (q < 10^-15.35^) are reported in this table as ‘***’ (< 0.001).

## 4 Discussion

The objective of this study was to characterize region-specific transcriptomic alterations across lesional, perilesional, and non-lesional skin in vitiligo, using minimally invasive 1 mm skin punch biopsies. We show that this approach yields high-quality transcriptomic data, captures both established and novel disease-associated gene expression signatures, and reveals candidate therapeutic pathways and signature-reversing compounds through data-driven analyses.

Consistent RNA yield and sequencing depth confirm that 1 mm punch biopsies support comprehensive, suture-free transcriptomic profiling in vitiligo. Despite their small size, they reliably capture core disease-related transcriptional programs, supporting a low-input, high-output workflow. While smaller samples may underrepresent broader tissue heterogeneity, their minimal invasiveness enables repeated, site-directed sampling and focused interrogation of local microenvironmental changes with high spatial specificity, establishing 1 mm biopsy transcriptomics as a practical, patient-friendly platform for molecular dermatology.

Although the use of 1 mm biopsies for transcriptomic profiling in inflammatory skin diseases remains limited, the available studies support the biological validity of this approach. Recent work in atopic dermatitis successfully used 1 mm punch biopsies for longitudinal skin RNA sequencing (14). In this study, large-scale transcriptomic profiling generated a high-quality dataset, with 98% of samples passing quality control, and recovered established disease-associated biology, including epidermal barrier and immunological dysfunction signatures consistent with prior studies. Complementary evidence comes from another atopic dermatitis study using 1.5 mm punch biopsies, in which the resulting full-thickness skin transcriptomes were shown to be comparable with those obtained from larger biopsies at the gene and pathway level (15). In addition, a psoriasis/eczema molecular classifier study demonstrated that 1 mm biopsies could recapitulate disease-specific RNA sequencing clustering and hallmark gene expression patterns observed in paired 4–6 mm biopsies, although with a small increase in misclassification in the minimally invasive samples (16). These data provide important methodological precedent that small punch biopsies can capture biologically informative skin transcriptomes in inflammatory dermatology, whereas directly comparable examples in vitiligo appear to be lacking. Beyond small punch biopsies, emerging microbiopsy-based strategies further highlight the field’s broader movement toward less invasive molecular skin sampling. At present, their use in inflammatory dermatology appears to remain at an early, predominantly methodological stage (17, 18). At the biological level, our data revealed a spatially organized pattern of transcriptional dysregulation across lesional, perilesional, and non-lesional vitiligo skin, consistent with distinct stages of disease involvement. The expected downregulation of melanogenesis-related genes in lesional skin, attenuating in perilesional skin and only partly retained in non-lesional skin, supports both sampling accuracy and the biological relevance of the regional profiles. Moreover, perilesional skin displayed upregulation of inflammatory transcripts such as *IFI44* and *IFI6*, consistent with perilesional skin being considered the zone of highest inflammatory activity in vitiligo (8). Alongside these interferon-inducible genes, we observed upregulation of *FOSB*, encoding a transcription factor responsive to stress and inflammatory cues that regulates cell proliferation, differentiation, and transformation (19). A recent study similarly identified *FOSB* as a dominant regulator in vitiligo-associated macrophages, further reinforcing its potential contribution to disease pathogenesis (20).

Across comparisons with healthy controls, lipid metabolism genes were consistently suppressed, supporting the recognized interplay between metabolic dysfunction and inflammatory signaling in vitiligo (21, 22). Additional novel findings point to previously unexplored mechanisms: downregulation of *PPP1R1A*, a protein phosphatase-1 (PP1) inhibitor, suggests altered PP1/T-cell receptor signaling, while upregulation of *FAT1* (linked to pro-inflammatory signaling in glioma) and *HORMAD1* (associated with NF-κB activation) further highlight inflammatory networks (23–25). Interestingly, no DEGs were detected between perilesional and non-lesional skin (P_adj_ < 0.05, |log_2_FC| ≥ 1), likely reflecting the 1 cm sampling proximity and the variable extension of the inflammatory border beyond visible depigmentation, which can blur molecular distinctions (26). These results indicate that nearby non-lesional skin already harbors early pathogenic signals, consistent with prior reports, and highlight non-lesional tissue as a potentially informative tissue region for biomarker discovery (9). When interpreting comparisons with healthy control skin, it should be noted that patient biopsies were obtained from the arm or leg, whereas control biopsies were consistently collected from the upper arm. As baseline skin transcriptional profiles may vary by anatomical site, a contribution of site-related heterogeneity to the patient-versus-control comparisons cannot be excluded.

### 4.1 Immune and metabolic pathways

At the immune level, pathway-level analysis recapitulated the well-established T helper 1/interferon gamma-driven inflammation of vitiligo skin. The positive enrichment of NOD-like receptor signaling and antigen-presentation pathways in both perilesional and non-lesional skin is likewise consistent with prior vitiligo studies, suggesting that these immune circuits remain active beyond visibly affected areas. Enrichment of neutrophil extracellular trap (NET) formation identifies a comparatively less characterized inflammatory process in vitiligo, with possible pathogenic relevance analogous to its reported autoimmune role in psoriasis (27, 28).

In parallel, negative enrichment of oxidative phosphorylation, retinol metabolism, and AMP-activated protein kinase (AMPK) signaling points to metabolic dysfunction in vitiligo skin. Since oxidative phosphorylation takes place in mitochondria, this finding is consistent with mitochondrial dysfunction and its correlated increased reactive oxygen species (ROS) production previously reported in vitiligo, promoting oxidative stress, melanocyte damage, and secondary immune activation (29). By contrast, our dataset highlighted reduced retinol metabolism and diminished AMPK signaling as less explored but biologically relevant metabolic alterations. Reduced retinol metabolism may reflect a loss of retinoid-mediated protection against oxidative stress and immune activation, whereas diminished AMPK activity may further increase melanocyte vulnerability through impaired autophagy support and reduced restraint of mammalian target of rapamycin complex 1 (mTORC1) (30, 31).

### 4.2 Cellular clearance and homeostasis pathways

Our analysis also identified altered expression of cellular clearance and homeostatic pathways, including ferroptosis, peroxisome, phagosome, and efferocytosis signaling. Although these pathways have received limited attention in vitiligo, they may be highly relevant to disease pathogenesis given their roles in apoptotic cell clearance, oxidative stress control, and immune regulation (32–36). Impaired activity of these pathways could hinder the removal of apoptotic or stressed cells, prolong antigen exposure, and thereby sustain local immune activation. These findings expand the current immune-centric view of vitiligo to encompass defects in tissue homeostasis and repair.

### 4.3 Cell growth and structural regulation

Pathways associated with cell growth and structural remodeling were also altered in vitiligo skin. The Hippo signaling pathway, which regulates cell proliferation, differentiation, and survival, was positively enriched in perilesional skin compared to controls (37). Dysregulation of this pathway has been implicated in immune dysfunction and melanocyte dedifferentiation, leading to loss of epidermal identity and impaired repigmentation (38). Its activation at sites of active inflammation in our dataset supports its role in melanocyte dysfunction and resistance to regenerative cues.

The extracellular matrix (ECM)–receptor pathway was positively enriched in lesional skin compared to both perilesional and control skin. It comprises molecules linking the ECM to the cytoskeleton and regulating adhesion, migration, and survival (39, 40). Contrary to previous reports of reduced integrin expression in vitiligo, our data showed no such negative enrichment (41). Instead, increased ECM–receptor signaling in lesional skin may reflect inflammatory remodeling, as several of these upregulated pathway genes such as *ITGAV* (log_2_FC = 0.460; P_adj_<0.001), *ITGA11* (log_2_FC = 0.450; P_adj_ > 0.05), *HMMR* (log_2_FC = 0.702; P_adj_ < 0.05), and *COMP* (log_2_FC = 1.778; P_adj_ < 0.001) are typically induced during chronic inflammation and tissue repair (42–44). This remodeling could coexist with local adhesion defects described in previous work, highlighting a more complex regulation of ECM interactions in vitiligo pathogenesis (41).

### 4.4 External validation against an independent bulk RNA sequencing vitiligo dataset

Targeted supplementary comparison with a recent bulk RNA sequencing vitiligo study provided additional external support for the biological fidelity of the present dataset (10). Despite limited overlap at the level of individual DEGs, both studies converged on the same disease-associated biological programs. This is biologically plausible, given that pathway-level analyses capture coordinated shifts across functionally related genes beyond those meeting strict DEG thresholds individually. Interestingly, inflammatory enrichment was most prominent in lesional and non-lesional skin in the study by Brunner et al. versus perilesional and non-lesional skin in the present study. Because of the spatially focused nature of a 1 mm biopsy, certain inflammatory programs may be detected more distinctly in the perilesional transition zone in our dataset, whereas a 5 mm biopsy averages over a larger and more heterogeneous tissue area and may therefore capture related signals within the lesional compartment itself. Collectively, these findings further support that the 1 mm biopsy approach preserves genuine vitiligo-associated biological programs while offering high spatial precision.

### 4.5 Mechanistically distinct candidate compounds point to multiple actionable pathways in vitiligo

The CMap analysis further contextualizes these transcriptional patterns in a therapeutic framework. Epigenetic modulators emerged as strong candidates, consistent with the positive enrichment of epigenetic silencing pathways in lesional skin in our dataset and with prior evidence implicating chromatin dysregulation in melanocyte apoptosis. Growth factor kinase inhibitors targeting EGFR, VEGFR, and related non-JAK tyrosine kinases emerged as additional hits, potentially linking these compounds to the inflammatory and immune-signaling pathways identified in our analyses through their known effects on cytokine-associated signaling. However, their use outside oncology remains constrained by dermatologic and vascular toxicities. ROCK inhibitors may similarly be of interest in vitiligo, as experimental studies suggest they can restore melanocyte dendricity and viability under inflammatory stress (45). Immune signaling modulators targeting BTK, SYK, and interleukin pathways further reflect interference with key inflammatory cascades, although compounds such as STA-5326 may also disrupt endolysosomal homeostasis. Likewise, metabolic modulators such as PDE and SphK inhibitors map onto the metabolic and oxidative stress dysregulation identified in the present study, while also being of interest because of their reported anti-inflammatory and pro-melanogenic effects. It should be noted that these predictions were derived from perturbational signatures in the A375 melanocytic cell line, which provides lineage relevance but does not recapitulate the multicellular complexity of vitiligo skin. Accordingly, the nominated compounds should be regarded as exploratory, hypothesis-generating candidates requiring validation in more disease-relevant experimental systems. Nevertheless, these results illustrate how small-sample transcriptomics has the potential to inform mechanism-based drug discovery, with possible future relevance for therapeutic stratification.

## 5 Conclusion

This study extends current understanding of vitiligo by both confirming established immunopathogenic signatures and uncovering underexplored immune, metabolic, and cellular clearance pathways across distinct skin regions. Using minimally invasive 1 mm punch biopsies, we show that small-sample profiling can capture biologically meaningful molecular differences across lesional, perilesional, and non-lesional skin. Connectivity Map analysis further nominated candidate pharmacological modulators, including epigenetic, kinase, metabolic and immune-targeted compounds, supporting the value of this approach for translational drug discovery in vitiligo.

## Data Availability

The datasets presented in this study can be found in online repositories. The names of the repository/repositories and accession number(s) can be found below: https://www.ncbi.nlm.nih.gov/geo/query/acc.cgi?acc=GSE311770.

## References

[1] Seneschal J . Clinical features of vitiligo and social impact on quality of life. Dermatol Pract Concept. (2023) 13:e2023312S. doi: 10.5826/DPC.1304S2A312S. PMID: 38241394 PMC10824319

[2] Speeckaert R Caelenberg E Van E Belpaire A Speeckaert MM van Geel N . Vitiligo: from pathogenesis to treatment. J Clin Med. (2024) 13. doi: 10.3390/JCM13175225. PMID: 39274437 PMC11396398

[3] Pathak GN Tan IJ Bai G Dhillon J Rao BK . Vitiligo: from mechanisms of disease to treatable pathways. Skin Health Dis. (2024) 4. doi: 10.1002/ski2.460. PMID: 39624766 PMC11608881

[4] Witek A Parys J Mikosińska A Kaźmierczak M Mossakowski M Kałuziak P . Vitiligo review: etiopathogenesis, diagnosis and treatment. Qual Sport. (2025) 37:57367. doi: 10.12775/QS.2025.37.57367

[5] Seremet T Di Domizio J Girardin A Yatim A Jenelten R Messina F . Immune modules to guide diagnosis and personalized treatment of inflammatory skin diseases. Nat Commun. (2024) 15:1–12. doi: 10.1038/s41467-024-54559-6. PMID: 39695162 PMC11655867

[6] Kalmar JR . Advances in the detection and diagnosis of oral precancerous and cancerous lesions. Oral Maxillofac Surg Clin North Am. (2006) 18:465–82. doi: 10.1016/j.coms.2006.06.013. PMID: 18088846

[7] Liu D Hu BD Mishra A Patel D Lau M Navrazhina K . Tape strips in inflammatory skin disease: a noninvasive method for molecular insights and personalized care. Br J Dermatol. (2025) 00:1–8. doi: 10.1093/BJD/LJAF275. PMID: 40658734

[8] Martins C Migayron L Drullion C Jacquemin C Lucchese F Rambert J . Vitiligo skin T cells are prone to produce type 1 and type 2 cytokines to induce melanocyte dysfunction and epidermal inflammatory response through Jak signaling. J Invest Dermatol. (2022) 142:1194–1205.e7. doi: 10.1016/J.JID.2021.09.015. PMID: 34655610

[9] Tulic MK Kovacs D Bastonini E Briganti S Passeron T Picardo M . Focusing on the dark side of the moon: involvement of the nonlesional skin in vitiligo. J Invest Dermatol. (2024) 145(7):1612–21. doi: 10.1016/J.JID.2024.10.598. PMID: 39708041

[10] Brunner PM David E Del Duca E Manson M Kurowski A Naidu MP . Transcriptomic profiling of vitiligo patients shows polar immune dysregulation in involved and uninvolved skin. J Allergy Clin Immunol. (2025) 156(4):993–1007. doi: 10.1016/J.JACI.2025.06.002. PMID: 40513622

[11] van Geel N Depaepe L Vandaele V Mertens L Van Causenbroeck J De Schepper S . Assessing the dynamic changes in vitiligo: reliability and validity of the vitiligo disease activity score (VDAS) and vitiligo disease improvement score (VDIS). J Eur Acad Dermatol Venereol. (2022) 36:1334–41. doi: 10.1111/JDV.18134. PMID: 35398942 PMC9543188

[12] Chen Y Chen L Lun ATL Baldoni PL Smyth GK . edgeR v4: powerful differential analysis of sequencing data with expanded functionality and improved support for small counts and larger datasets. Nucleic Acids Res. (2025) 53. doi: 10.1093/NAR/GKAF018. PMID: 39844453 PMC11754124

[13] Yu G Wang LG Han Y He QY . clusterProfiler: an R package for comparing biological themes among gene clusters. OMICS. (2012) 16:284–7. doi: 10.1089/OMI.2011.0118. PMID: 22455463 PMC3339379

[14] Fukushima-Nomura A Kawasaki H Yashiro K Obata S Tanese K Ebihara T . An unbiased tissue transcriptome analysis identifies potential markers for skin phenotypes and therapeutic responses in atopic dermatitis. Nat Commun. (2025) 16:4981–. doi: 10.1038/s41467-025-59340-x. PMID: 40456762 PMC12130345

[15] Hu T Todberg T Ewald DA Hoof I Correa da Rosa J Skov L . Assessment of spatial and temporal variation in the skin transcriptome of atopic dermatitis by use of 1.5 mm minipunch biopsies. J Invest Dermatol. (2023) 143:612–620.e6. doi: 10.1016/j.jid.2022.10.004. PMID: 36496193

[16] Fischer F Doll A Uereyener D Roenneberg S Hillig C Weber L . Gene expression–based molecular test as diagnostic aid for the differential diagnosis of psoriasis and eczema in formalin-fixed and paraffin-embedded tissue, microbiopsies, and tape strips. J Invest Dermatol. (2023) 143:1461–1469.e5. doi: 10.1016/J.JID.2023.02.015. PMID: 36889660

[17] Lei BUW Yamada M Hoang VLT Belt PJ Moore MH Lin LL . Absorbent microbiopsy sampling and RNA extraction for minimally invasive, simultaneous blood and skin analysis. J Vis Exp. (2019) 2019. doi: 10.3791/58614. PMID: 30855573

[18] Kislevitz M Wamsley C Bartels M Lu KB Li X Pinch S . Clinical translation of scarless 0.33-mm core microbiopsy for molecular evaluation of human skin. Aesthet Surg J. (2021) 41:NP1710–20. doi: 10.1093/ASJ/SJAA332. PMID: 33252635

[19] FOSB FosB proto-oncogene, AP-1 transcription factor subunit [Homo sapiens (human)] - Gene - NCBI. Available online at: https://www.ncbi.nlm.nih.gov/gene?Db=gene&Cmd=ShowDetailView&TermToSearch=2354.

[20] Yu Y Wang Y Lu J Cao X Feng Y Pei T . A comparative analysis dissecting the immune landscape of vitiligo and melanoma from a single-cell perspective: two sides of the same coin? Inflammation. (2025) 47:1–19. doi: 10.1007/s10753-025-02332-2 PMC12722509 40608219

[21] Glass CK Olefsky JM . Inflammation and lipid signaling in the etiology of insulin resistance. Cell Metab. (2012) 15:635–45. doi: 10.1016/j.cmet.2012.04.001. PMID: 22560216 PMC4156155

[22] Lyu C Sun Y . Immunometabolism in the pathogenesis of vitiligo. Front Immunol. (2022) 13:1055958. doi: 10.3389/fimmu.2022.1055958 36439174 PMC9684661

[23] Wu CS Liu FC Lin SC Chyuan IT . Regulation of T cell receptor (TCR) signaling by tyrosine phosphatases: recent advances and implication for therapeutic approach in autoimmune diseases. J Formosan Med Assoc. (2025). doi: 10.1016/J.JFMA.2025.04.023. PMID: 40287371

[24] Arora M Kundu A Sinha S Chosdol K . Immune factors and their role in tumor aggressiveness in glioblastoma: atypical cadherin FAT1 as a promising target for combating immune evasion. Cell Mol Biol Lett. (2025) 30:89. doi: 10.1186/S11658-025-00769-9. PMID: 40713501 PMC12291518

[25] Liu K Cheng L Zhu K Wang J Shu Q . The cancer/testis antigen HORMAD1 mediates epithelial-mesenchymal transition to promote tumor growth and metastasis by activating the Wnt/β-catenin signaling pathway in lung cancer. Cell Death Discov. (2022) 8:136. doi: 10.1038/s41420-022-00946-1. PMID: 35347116 PMC8960869

[26] Aslanian FMNP Noe RAM Antelo DP Farias RE Das PK Galadari I . Immunohistochemical findings in active vitiligo including depigmenting lesions and non-lesional skin. Open Dermatol J. (2008) 2:105–10. doi: 10.2174/1874372200802010105

[27] Shao S Fang H Dang E Xue K Zhang J Li B . Neutrophil extracellular traps promote inflammatory responses in psoriasis via activating epidermal TLR4/IL-36R crosstalk. Front Immunol. (2019) 10:746. doi: 10.3389/fimmu.2019.00746 31024570 PMC6460719

[28] Wang H Kim SJ Lei Y Wang S Wang H Huang H . Neutrophil extracellular traps in homeostasis and disease. Signal Transd Targ Ther. (2024) 9:1–40. doi: 10.1038/s41392-024-01933-x. PMID: 39300084 PMC11415080

[29] Lin Y Ding Y Wu Y Yang Y Liu Z Xiang L . The underestimated role of mitochondria in vitiligo: from oxidative stress to inflammation and cell death. Exp Dermatol. (2024) 33:e14856. doi: 10.1111/EXD.14856. PMID: 37338012

[30] Li G Qu B Zheng T Cheng Y Li P Liu Z . Assessing the causal effect of genetically predicted metabolites and metabolic pathways on vitiligo: evidence from Mendelian randomization and animal experiments. J Steroid Biochem Mol Biol. (2025) 247:106677. doi: 10.1016/J.JSBMB.2025.106677. PMID: 39818343

[31] Bastonini E Kovacs D Raffa S delle Macchie M Pacifico A Iacovelli P . A protective role for autophagy in vitiligo. Cell Death Dis. (2021) 12:1–15. doi: 10.1038/s41419-021-03592-0. PMID: 33767135 PMC7994839

[32] Mantegazza AR Magalhaes JG Amigorena S Marks MS . Presentation of phagocytosed antigens by MHC class I and II. Traffic. (2012) 14:135. doi: 10.1111/TRA.12026. PMID: 23127154 PMC3538944

[33] Tiew TWY Sheahan MB Rose RJ . Peroxisomes contribute to reactive oxygen species homeostasis and cell division induction in Arabidopsis protoplasts. Front Plant Sci. (2015) 6:658. doi: 10.3389/fpls.2015.00658 26379686 PMC4549554

[34] Meghnem D Leong E Pinelli M Marshall JS Di Cara F . Peroxisomes regulate cellular free fatty acids to modulate mast cell TLR2, TLR4, and IgE-mediated activation. Front Cell Dev Biol. (2022) 10:856243. doi: 10.3389/fcell.2022.856243 35756999 PMC9215104

[35] Li J Cao F Yin H Huang Z Lin Z Mao N . Ferroptosis: past, present and future. Cell Death Dis. (2020) 11:1–13. doi: 10.1038/s41419-020-2298-2. PMID: 32015325 PMC6997353

[36] Ge Y Huang M Yao YM . Efferocytosis and its role in inflammatory disorders. Front Cell Dev Biol. (2022) 10:839248. doi: 10.3389/fcell.2022.839248. PMID: 35281078 PMC8913510

[37] Fu M Hu Y Lan T Guan KL Luo T Luo M . The Hippo signalling pathway and its implications in human health and diseases. Signal Transd Targ Ther. (2022) 7:1–20. doi: 10.1038/s41392-022-01191-9. PMID: 36347846 PMC9643504

[38] Yang F Yang L Lai S Yokota M Kuroda Y Yuki T . Aberrant laminin signaling drives melanocyte dedifferentiation and unveils a tractable therapeutic target in vitiligo. BioRxiv. (2025), 2025.04.11.648350. doi: 10.1101/2025.04.11.648350. PMID: 41997929 PMC13272753

[39] KEGG PATHWAY: ECM-receptor interaction - Homo sapiens (human) n.d. Available online at: https://www.kegg.jp/pathway/hsa04512 (Accessed September 4, 2025).

[40] Yue B . Biology of the extracellular matrix: an overview. J Glaucoma. (2014) 23:S20. doi: 10.1097/IJG.0000000000000108. PMID: 25275899 PMC4185430

[41] Su M Yi H He X Luo L Jiang S Shi Y . miR-9 regulates melanocytes adhesion and migration during vitiligo repigmentation induced by UVB treatment. Exp Cell Res. (2019) 384. doi: 10.1016/j.yexcr.2019.111615. PMID: 31499059

[42] Schulz JN Zeltz C Sørensen IW Barczyk M Carracedo S Hallinger R . Reduced granulation tissue and wound strength in the absence of α11β1 integrin. J Invest Dermatol. (2015) 135:1435–44. doi: 10.1038/JID.2015.24. PMID: 25634355 PMC4407012

[43] Jaskuła K Sacharczuk M Gaciong Z Skiba DS . Cardiovascular effects mediated by HMMR and CD44. Mediators Inflammation. (2021) 2021:4977209. doi: 10.1155/2021/4977209. PMID: 34335086 PMC8286199

[44] Carlsén S Hansson AS Olsson H Heinegård D Holmdahl R . Cartilage oligomeric matrix protein (COMP)-induced arthritis in rats. Clin Exp Immunol. (2001) 114:477–84. doi: 10.1046/J.1365-2249.1998.00739.X. PMID: 9844060 PMC1905143

[45] Komatsu T Dong Y Ikeda T Kawakami T . Linking IFN-γ-mediated pathogenesis to ROCK-targeted therapy in a scalable iPSCs-based vitiligo model. Int J Mol Sci. (2025) 26. doi: 10.3390/IJMS26168069. PMID: 40869390 PMC12386722

