# Supplementary material for: Minimally invasive 1 mm skin biopsies capture site-specific transcriptomic heterogeneity in vitiligo
Source: Front Immunol. 2026 May 14;17:1829996. doi: 10.3389/fimmu.2026.1829996 (PMC13216050; doi:10.3389/fimmu.2026.1829996)
Supplement: Supplementary file 1 [file DataSheet1.zip › Supplementary_Figure S1_S3.docx]

**Supplementary figures**


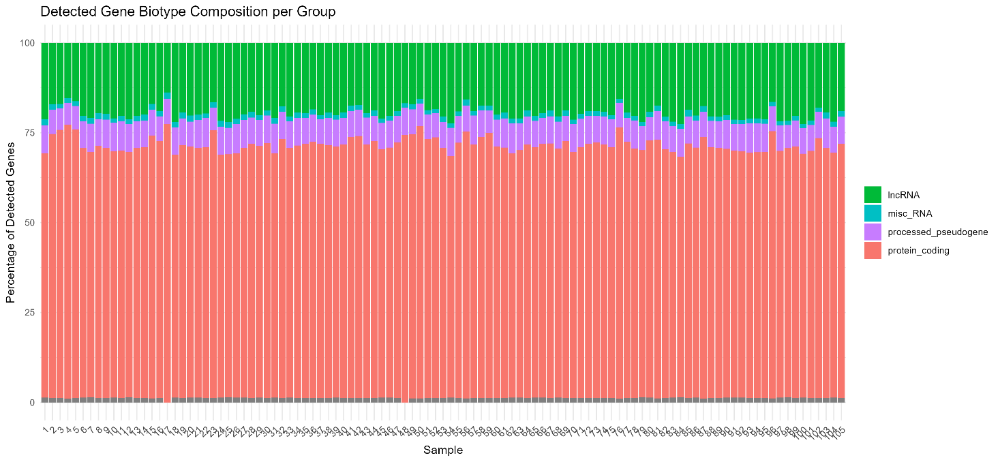


**Figure S1.** Relative composition of detected gene biotypes per sample. Stacked barplots display the proportion of protein-coding, lncRNA, and other non-coding gene types based on the obtained count table. On average, 15,012 protein-coding genes were detected per sample, representing the majority of mapped features.


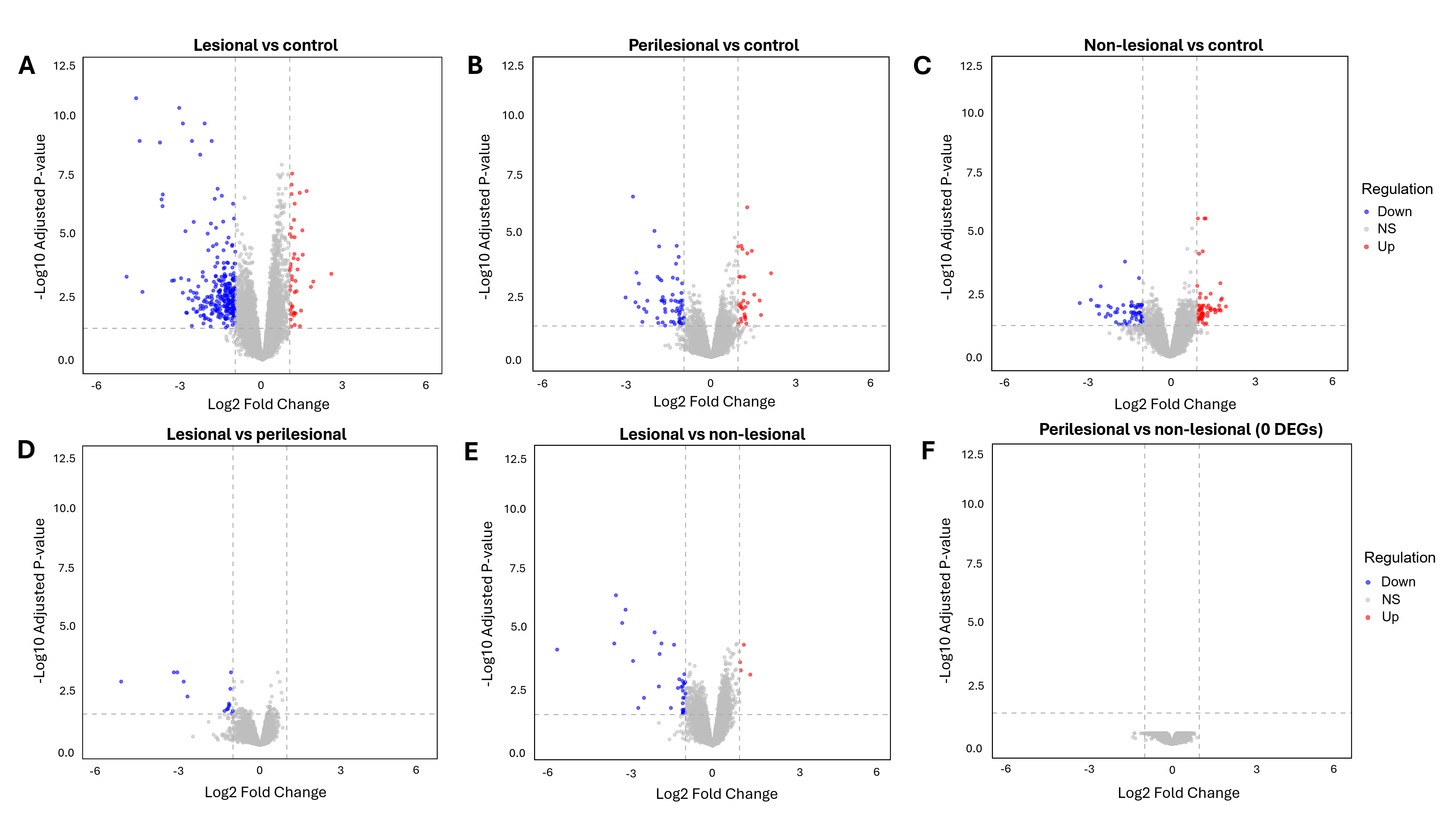


**Figure S2.** Each panel shows a pairwise comparison between skin regions from vitiligo patients and healthy controls: (A) Lesional (n = 24) vs healthy control (n = 27), (B) Perilesional (n = 27) vs healthy control (n = 27), (C) Non-lesional (n = 27) vs healthy control (n = 27), (D) Lesional (n = 24) vs perilesional (n = 27), (E) Lesional (n = 24) vs non-lesional (n = 27) and (F) Perilesional (n = 27) vs non-lesional (n = 27). Genes with adjusted p-value < 0.05 and |log₂ fold change| ≥ 1 are highlighted in red (upregulated) or blue (downregulated), while non-significant genes are shown in grey. The perilesional versus non-lesional comparison (panel F) showed no significant differentially expressed genes at the applied thresholds. Axes represent log₂ fold change (x-axis) and –log₁₀ adjusted p-value (y-axis). The same plotting thresholds were applied across all volcano plots to facilitate direct visual comparison between contrasts. NS, Not significant; DEGs, differentially expressed genes.


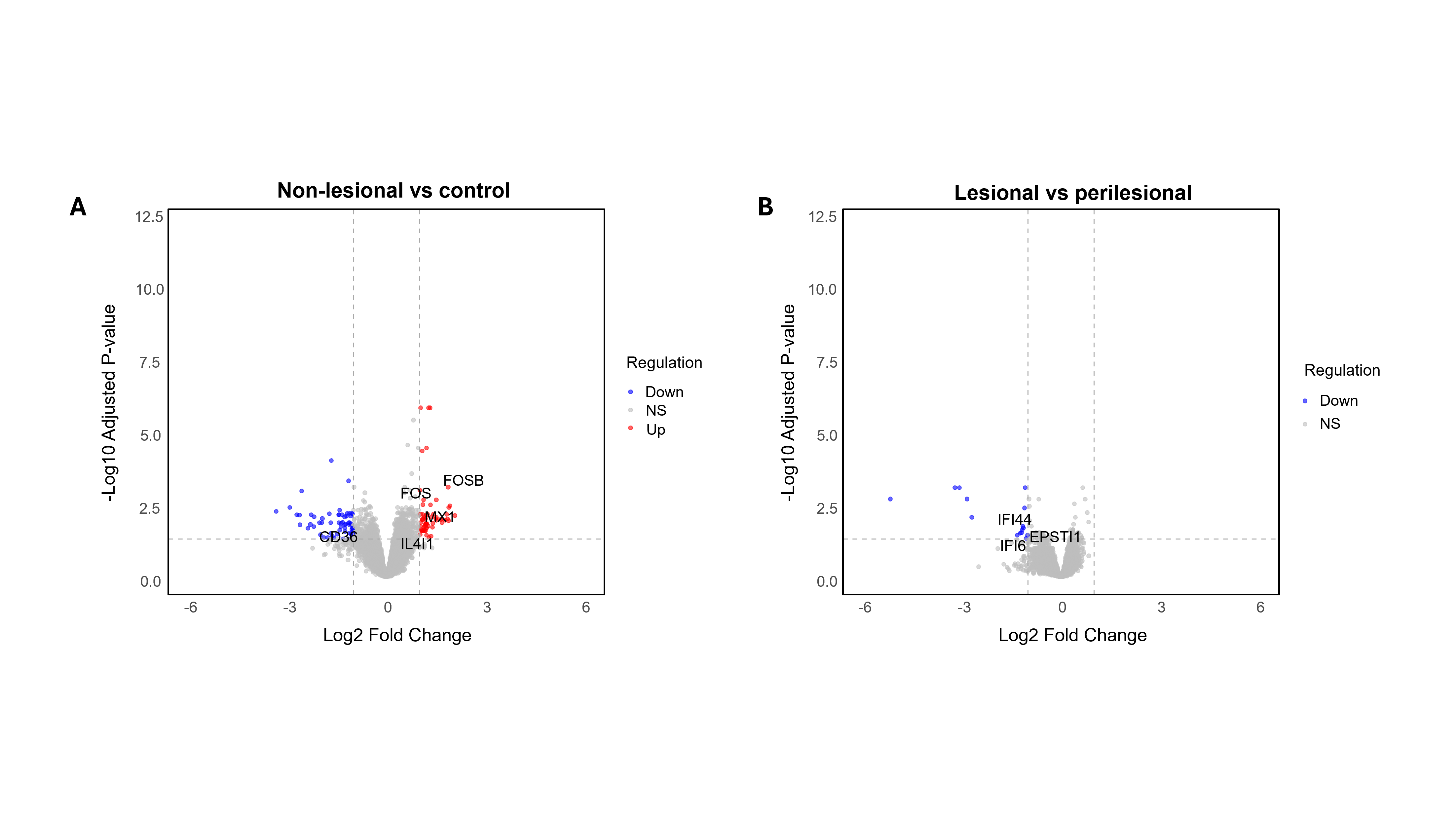


**Figure S3.** Volcano plots highlighting immune-related transcriptional changes in vitiligo skin. Volcano plots display differentially expressed genes in (A) Non-lesional (n = 27) vs healthy control (n = 27) and (B) Lesional (n = 24) vs perilesional skin (n = 27). Genes with adjusted p-value < 0.05 and |log₂ fold change| ≥ 1 are highlighted in red (upregulated) or blue (downregulated), while non-significant genes are shown in grey. Axes represent log₂ fold change (x-axis) and –log₁₀ adjusted p-value (y-axis). The same plotting thresholds were applied across all volcano plots to facilitate direct visual comparison between contrasts. Immune-related genes including cytokines, chemokines, interferon-stimulated genes, transcription factors, and immune modulators, are annotated where differentially expressed. NS, Not significant.
